# Severely stigmatised skin neglected tropical diseases: a protocol for social science engagement

**DOI:** 10.1093/trstmh/traa141

**Published:** 2020-12-16

**Authors:** Shahaduz Zaman, Papreen Nahar, Hayley MacGregor, Tom Barker, Jeannette Bayisenge, Clare Callow, James Fairhead, Ahmed Fahal, Natalia Hounsome, Anne Roemer-Mahler, Peter Mugume, Getnet Tadele, Gail Davey

**Affiliations:** Centre for Global Health Research, Brighton and Sussex Medical School, University of Sussex, Brighton, BN1 9PX, UK; Centre for Global Health Research, Brighton and Sussex Medical School, University of Sussex, Brighton, BN1 9PX, UK; Institute of Development Studies, University of Sussex, Brighton, BN1 9RE, UK; Institute of Development Studies, University of Sussex, Brighton, BN1 9RE, UK; University of Rwanda, Kigali, POB 4285, Rwanda; Centre for Global Health Research, Brighton and Sussex Medical School, University of Sussex, Brighton, BN1 9PX, UK; School of Global Studies, University of Sussex, Brighton, BN1 9S, UK; Mycetoma Research Centre, University of Khartoum, Khartoum, POB 102, Sudan; Centre for Global Health Research, Brighton and Sussex Medical School, University of Sussex, Brighton, BN1 9PX, UK; School of Global Studies, University of Sussex, Brighton, BN1 9S, UK; University of Rwanda, Kigali, POB 4285, Rwanda; College of Social Sciences, Addis Ababa University, Addis Ababa, Ethiopia; Centre for Global Health Research, Brighton and Sussex Medical School, University of Sussex, Brighton, BN1 9PX, UK

**Keywords:** NTD, skin disease, social science, stigma

## Abstract

More than one billion people are affected by neglected tropical diseases (NTDs) and many of these diseases are preventable. While the grouping of these conditions as NTDs has generated vast mapping, mass drug administration and surveillance programmes, there is growing evidence of gaps and weaknesses in purely biomedical approaches, and the need for responses that also recognise the social determinants of health. In order to unpack the social and political determinants of NTDs, it is important to view the problem from a social science perspective. Given this background, the Social Sciences for Severe Stigmatizing Skin Diseases (5S) Foundation has recently been established by the Centre for Global Health Research at Brighton and Sussex Medical School. The broad aim of the 5S Foundation is to incorporate social science perspectives in understanding and addressing the problems around three NTDs, namely, podoconiosis, mycetoma and scabies. This protocol paper sets out the aims and approaches of the 5S Foundation while activities such as research, public engagement, training and capacity building get underway.

## Introduction

Neglected tropical diseases (NTDs) are a diverse group of diseases that prevail in tropical and subtropical conditions and affect more than one billion people.^1^ Populations living in poverty, without adequate sanitation and in close contact with infectious vectors and domestic animals or livestock, are those which are worst affected. In addition to their impact on health, NTDs contribute to an immense social and economic burden resulting in social stigma, physical disabilities, disfigurement, discrimination, loss of social status, malnutrition, growth failure and impaired cognitive development.^1,2^ The morbidity and suffering also depend on access to healthcare as paying out of pocket for healthcare is common in many settings where NTDs are prevalent. These social, economic and physical consequences prevent individuals from leading productive lives and ultimately trap them in a cycle of poverty. In the 2017 Global Burden of Disease Study, NTDs accounted for 62.3 million disability-adjusted life years.^3^

However, many of these diseases are preventable, and could be eliminated with improved sanitation, vector control, available treatments and mass drug administration (MDA), when treatment is provided to every member of a defined population or every person living in a defined geographical area at approximately the same time and often at repeated intervals. Although these conditions are preventable, in global health, interventions which may have a strong evidence base and appear straightforward to implement often encounter unexpected barriers in real life. It is well acknowledged that for a health intervention to be successful, it is important to pay attention to the social determinants of health, which are the conditions in which people are born, grow, work, live and age, and the wider set of forces and systems shaping the conditions of daily life including how institutions, interests and power-relations shape health outcomes.^4^ There is growing awareness that global health is a biosocial issue that requires a biosocial approach. Authors have highlighted how the clinical, biomedical and epidemiological disciplinary perspectives are dominant in wider health research.^5^ They argue that health policies and systems are complex social and political phenomena, constructed by human action rather than occurring naturally. Relativist social science perspectives are, therefore, of particular relevance to health policy and system research as they recognise that all phenomena are in essence constructed through human behaviour and interpretation. Advancing the science of health research thus demands that we take steps to build understanding across disciplinary boundaries.

Within the field of NTDs, a clear example of the need for a biosocial approach is the low coverage of populations that would benefit from regular treatment to prevent disease. The ‘gap’ between knowledge and practice is also large when behavioural changes are necessary, whether this is face-washing to prevent the eye disease trachoma or shoe-wearing to prevent podoconiosis.^6^ However, it is important to acknowledge that such behavioural changes are affected not only by knowledge but also by structural conditions. In order to unpack the social and political determinants of NTDs it is important to view the problem from a social science perspective. Social sciences can provide theoretical insights and methodological approaches to analyse the framing of programmes and interventions as well as to make interventions contextually sensitive.^7^ However, in comparison with the biomedical disciplines, the social sciences have been underutilised in relation to NTDs.^8,9^ The term ‘second translational gap’ has been used to describe this knowledge-practice disconnect in public health in the Global North,^10^ but it is found worldwide.

Political economy is broadly defined as the study of both politics and economics, specifically the interactions between them and their consequences for specific outcomes of interest.^11^ Political economy of health refers to analysis of the conditions that shape population health and health service development within the wider macroeconomic and political context. Various authors have offered a critique of the framing of interventions for NTDs and the way in which an emphasis on behavioural change to the exclusion of an examination of political economy can inadvertently stigmatise local people. The political economy critique has also been extended to MDA programmes and the way in which these have been linked to development goals and ‘ending poverty’. These studies have documented how disjunctures between the biomedical and local understandings of NTDs and historical experiences of public health interventions have led to ‘resistance’ in African settings. The authors argue that without social, political and culturally sensitive strategies and greater attention to the way in which global health metrics are employed, mass drug treatment and other interventions faces challenges in local settings and national programme targets might be divorced from realities and experiences on the ground.^12-14^ In addition to these gaps at individual and community levels, the lack of national and international level policy research hinders policy development and advocacy attempts. The notion of ‘neglect’ in the term NTD has also been contested from the point of view of ‘political economy of the soil’.^15^

Given this background, the Social Sciences for Severe Stigmatizing Skin Diseases (5S) Foundation has recently been established by the Centre for Global Health Research at Brighton and Sussex Medical School with a 4-y grant from the National Institute of Health Research (2019–2023). The term ‘social sciences’ is used here to cover a range of academic disciplines that are broadly concerned with human society. Several of these disciplines, including anthropology, medical sociology, social policy, political science, social epidemiology and economics, are of great relevance for global health. The broad aim of the 5S Foundation is to incorporate social science perspectives in understanding and addressing the problems around three NTDs, namely, podoconiosis, mycetoma and scabies, which attract unfair community disapproval (‘stigma’). Podoconiosis is a progressive, debilitating form of leg swelling experienced by barefoot farmers; mycetoma is a slow-growing, destructive infection of the skin and underlying tissues; and scabies is an extremely itchy infectious condition caused by skin-burrowing mites. These conditions are huge public health problems in the three countries in which the 5S Foundation will work, namely, Ethiopia, Sudan and Rwanda.

Previous studies of health-related stigma, including stigma-reducing interventions in Africa, have been focused on HIV,^16-18^ mental health,^19^ epilepsy,^20^ TB,^21^ diabetes^22^ and leprosy.^23^ These studies have aimed at identifying social, cultural, political and economic predictors of stigma,^16,23^ evaluating interventions for stigmatised conditions,^17,21^ assessing government programmes to reduce stigma^19^ and scoping for new interventions.^21^ Stigma studies of NTDs covered by the scope of the 5S Foundation are rare (e.g. podoconiosis)^23^ or do not exist (mycetoma and scabies). The 5S Foundation will take advantage of a multidisciplinary team to examine cultural, social and economic contexts of podoconiosis, mycetoma and scabies to investigate how these diseases have been conceptualised at the national and international policy level, to identify existing interventions for stigmatised skin diseases, to develop comprehensive intervention strategies for the three NTDs and to strengthen the capacity for social science research in Ethiopia, Sudan and Rwanda.

This protocol paper sets out the aims and approaches of the 5S Foundation while activities such as research, public engagement, training and capacity building get underway.

## Vision of the 5S Foundation

The vision of the 5S Foundation is to end neglect apparent at three levels: neglect of conditions, countries and disciplines. In more detail, this applies to (1) neglect of three heavily stigmatising skin conditions: podoconiosis, mycetoma and scabies; (2) neglect of affected patients and communities at different levels in three countries: Ethiopia, Sudan and Rwanda; and (3) neglect of the social sciences as vital global health disciplines through interdisciplinary capacity strengthening in the aforementioned countries.

Each of the conditions mentioned above is hugely neglected in terms of research, intervention and local, national and global priority.^24,25^ The 5S Foundation aims to conduct research that will contribute to changes that will improve the health and well-being of people affected by these three conditions by identifying interventions informed by social science perspectives at the level of the patient, the community and national policy in the three selected countries in which the Foundation is working. These countries have been selected on the basis of the significant burden of NTDS in these three countries, existing partnerships and high priority needs related to the three NTDs that the Centre for Global Health Research focuses on. For instance, Ethiopia bears the highest burden of podoconiosis in the world,^26^ and experienced one of the largest scabies outbreaks ever documented during 2014–2016.^27^ Sudan bears the highest burden of mycetoma globally^28^ and the Mycetoma Research Centre is the sole referral centre for the condition in the country and the only WHO Collaborating Centre on Mycetoma. Rwanda now has evidence of people affected with podoconiosis in all 30 districts.^29^ Furthermore, there are pragmatic reasons for selecting these countries because Brighton and Sussex Medical School has an existing network with these countries.

Although research by social scientists has uncovered important knowledge about podoconiosis, predominantly at the level of the individual, community and health system, very limited research of this nature has been conducted in relation to scabies and mycetoma.^30,31^ Studies on podoconiosis have addressed cultural belief systems, the use of footwear and stigma.^32^^–^^36^ However, very little research has been conducted in relation to any of these conditions at national or global levels, the levels at which health systems and policies are planned.

The research, as part of this foundation, will also focus on exploring broader societal factors that might shape disease experience or responses to social exclusion or different levels of disability. In the respective countries, these might include factors such as the privatisation of healthcare, existence of health insurance and the role of non-profit organisations (NGOs). This is also to note that all three partner countries have been affected by internal conflict. This historical fact has also affected the health systems of the target countries.

## Aims of the 5S Foundation

The 5S Foundation's specific aims can be grouped into situational, strategic and capacity-building aims, as outlined below.

### Situational aims

1. To examine the cultural logics and social, political and economic contexts of mycetoma, podoconiosis and scabies, utilising cross-cutting social science perspectives;
2. To understand the individual experience of the affected persons and the dynamics and dimensions of stigma at community level;
3. To investigate how all three diseases have been conceptualised in national and international policies, including within the broader framing of NTDs;
4. To evaluate existing NTD-related community interventions.

### Strategic aims

1. To refine a framework developed to identify gaps and potentially effective intervention packages to contribute to changes (at micro, meso and macro level) that will improve the health and well-being of people affected by podoconiosis, mycetoma and scabies in the three selected countries;
2. To develop a comprehensive intervention strategy for each disease utilising all the evidence gathered from the above.

### Capacity-building aims

1. To support endemic-country training posts (PhD and postdoctoral) in a manner that will leave enduring capacity for social science research across a range of local health priorities, including NTDs;
2. To facilitate South-South and North-South collaboration of best practice in research and advocacy applicable to a wide range of stigmatising conditions in low-economic resource and low-literacy settings.

## Conceptual framework

**Figure 1.**
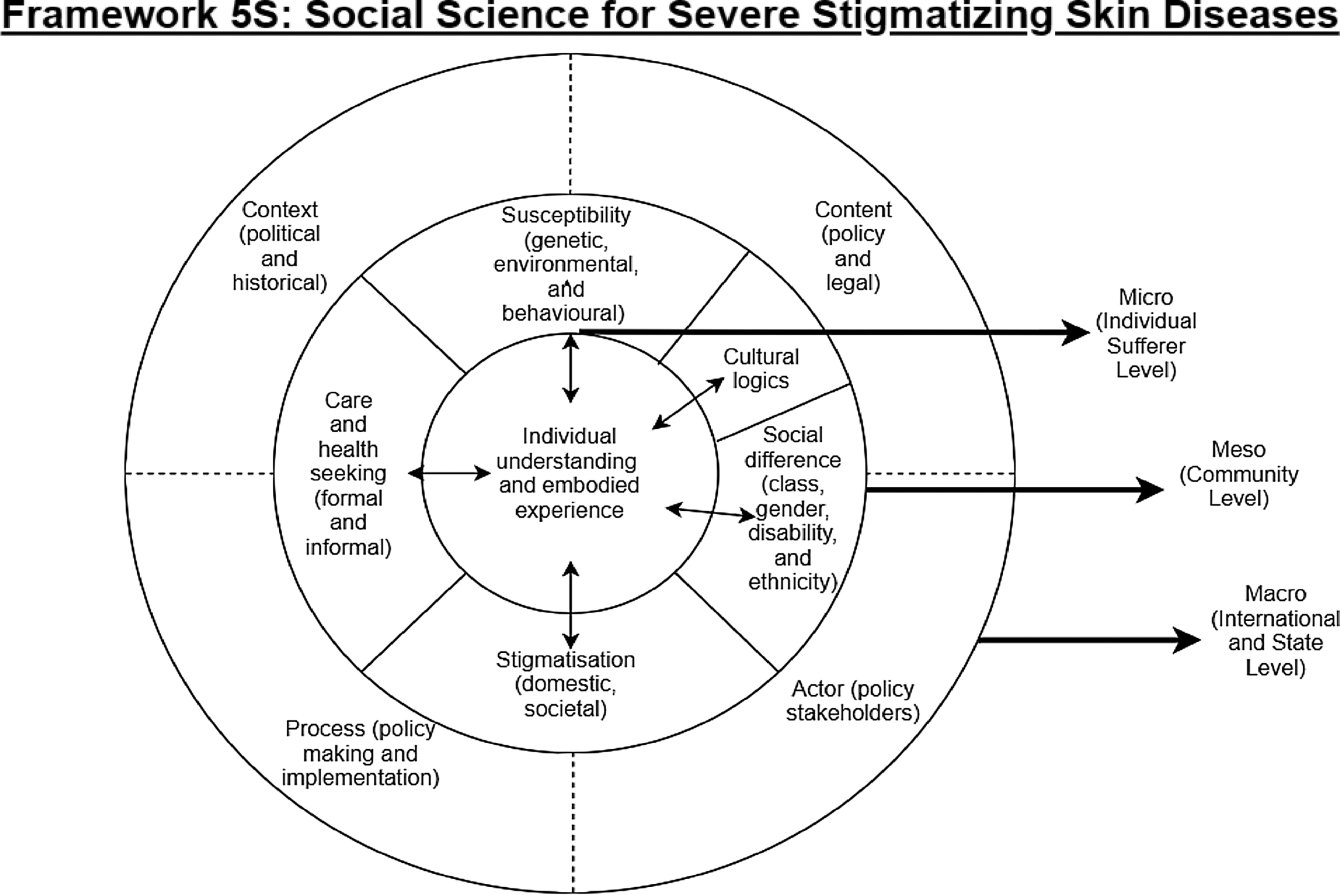
Framework of the 5S Foundation.

The 5S Foundation has developed a conceptual framework to guide social science research for NTDs in general and severely stigmatised disease in particular. Ideas from eco-social theory,^37^ the Framework Integrating Normative Influences on Stigma^38^ and discussions around multiple perspectives in health policy and systems research^39,40^ have been borrowed to develop this framework. As demonstrated in Figure 1 below, the framework uses three levels, ‘macro’, ‘meso’ and ‘micro’, presented as concentric circles, broadly covering illness experience, health services and the political and policy arena. This is to point out that while we will follow an inductive approach to social science research, we believe a framework is useful to guide a large multi-country, multi-disciplinary project like the 5S Foundation. While the fieldwork may raise new themes and unexpected findings, this broad conceptual framework distils some of the key areas that we think will need to be considered.

## Approaches to address the situational aims

In order to address the situational aims, data will be collected at three levels using a range of methods as described below. Although the choice of the study approaches will depend on the topic of the study, broadly grounded theory and phenomenological approaches will be applied. Grounded theory sets out to discover or construct theory from data, systematically obtained and analysed using comparative analysis.^41^ On the other hand, phenomenology is a form of qualitative research that focuses on the study of an individual's lived experiences within the world.^42^

### Macro-level situation:

Two sets of data will be collected from the macro or national level.

(1) Existing health systems, facilities and interventions

Using a semi-structured template, information about existing country-specific health systems and facilities will be collected from secondary sources and, if needed, primary sources through key informant interviews with relevant stakeholders.

(2) Current health policy environment

This will involve the exploration of the policy environment in relation to NTDs internationally and within each partner country. The policy environment will be explored using the policy analysis triangle.^39^ Data will be collected on the following four issues:

Context: To explore the social, historical, economic and political context of health-related policies in each country.

Content: To analyse the existence and content of relevant policies and strategies around the three skin diseases at national and international levels, and to examine how these diseases have been conceptualised.

Actors: To identify the key actors and their activities in the health domain in the respective countries. This will include a wide range of national, international, government and non-government stakeholders.

Process: To examine the process of policy-making and implementation, in particular, the roles of research and other national and international agendas.

Data sources: Policy environment-related data will be obtained from primary and secondary sources. Secondary sources include documents on national and international health (and other) policies, strategies and guidelines; journal articles; grey literature (including reports, newspapers and blogs); and any other organisational or archival documents that shed light on the social, historical and political contexts of the above. Primary sources of information about the policy-making and implementation processes in each country will include policy-makers and other key decision-makers within public, private and NGO domains. Methods will include document analysis (secondary data) and key informant interviews with primary sources.

### Meso-level situation

At the meso-level, two sets of data will be collected:

(1) Community members’ conceptualisation of the target diseases. We acknowledge that any given community might have plural views. This will be explored by investigating a range of themes including:

Cultural logics: What are the local names for the target diseases, and what are the meanings and myths?

Social difference: How does social difference, in terms of class, gender, disability and ethnicity, influence the experience of the target diseases?

Stigmatisation: What is the nature and level of stigma attached to the target diseases? Stigma would be a core theme of this project. We will explore family- and community-level stigma as well as possible stigma generated through ‘systematic exclusion’ by state policies. Goffman's pioneering idea of ‘spoiled identity’ will help us in understanding the basic tenets of social stigma. For him, stigma is an attribute, behaviour or reputation that is socially discrediting in a particular way: it causes an individual to be mentally classified by others in an undesirable, rejected stereotype rather than in an accepted, normal one.^43^ However, over the decade the concept of stigma has been expanded. More contemporary authors have critiqued the individually focused understanding of stigma and defined stigma as the co-occurrence of its components, labelling, stereotyping, separation, status loss and discrimination.^44^ They further indicated that for stigmatisation to occur, power must be exercised.

Care of the patient: What are the formal and informal sources of care available for those affected by the target skin conditions?

Susceptibility: What are the genetic, environmental and behavioural susceptibilities to the target diseases?

Meso-level data will be collected from community members and leaders and from formal and informal health and social care providers in urban and rural settings of the partner countries, including healthcare settings. Data will be gathered through focus group discussions, key informant interviews, focused ethnography and record review (to glean economic data for cost analysis of existing interventions).

Unlike traditional ethnography, in focused ethnography researchers enter the field with specified research questions and collect data through participant observation in the natural setting of the study population.^45^

(2) Evaluation of community interventions

At the meso-level, existing community interventions against the target diseases will be evaluated. The interventions may be of various natures including public health interventions or education interventions. As part of the macro-level situation analysis, we will list all the existing community engagement activities in the respective countries and then select one for evaluation. We are aware of various community engagement activities including social media campaigns, community theatre and other forms of public engagement like ‘community conversation through coffee ceremony’ in the target countries. A mixed methods evaluation framework is being developed to carry out the evaluation in each country. One dedicated postdoctoral research fellow will be responsible for carrying out the evaluation in each country. Two community interventions have been initially identified for evaluation. These are two rehabilitation projects, one in Rwanda for podoconiosis patients and the other in Sudan for mycetoma patients. The rehabilitation projects provide various livelihood supports to the affected persons.

### Micro-level situation

Two sets of data will be collected:

1. Individual-level embodied experience of the NTD-affected person will be explored. This will include:
  - The affected person's understandings of the causes of the NTD and their management of the condition;
  - Health-seeking behaviour of the affected person and responses to the disease;
  - Dynamics and dimensions of stigma experienced by the affected person;
  - Social and emotional consequences of the disease on the affected person's life.
2. Caregivers’ experience at the household level:
  - The factors that inhibit or encourage caregiving at the household level;
  - Household economic costing in relation to the disease.

Data will be gathered from adults and children affected by mycetoma, podoconiosis and scabies, and their household-level caregivers using life history and in-depth interview methods, as well as a semi-structured economic costing questionnaire.

## Approaches to address the strategic aims

The data collected through macro-, meso- and micro-level situation analysis will provide us with insight into the following issues:

- Gaps within and between the different levels;
- Factors enabling and disabling interventions to enhance the well-being of affected persons;
- The challenges of conducting projects to address severely stigmatised diseases.

The last will also be investigated through a process evaluation (explained in the following section).

Based on the evidence gathered, we will refine the framework developed earlier and finally propose an intervention strategy for each disease. Some of these interventions will be adopted as components of the 5S Foundation's impact strategy (Table 1).

**Table 1.** Summary of the methodology

Level of data	Method	Source of data
Macro-level: This will involve the exploration of the policy environment in relation to NTDs internationally and within each partner country	1. Documents analysis2. Key informant interviews	Documents on national and international health policies, strategies and guidelines; journal articles; grey literaturePolicy-makers and other key decision-makers within public, private and NGO domains
Meso-level: Community members’ conceptualisation of the target diseases	Focus group discussions, key informant interviews, focused ethnography (to observe community members’ interactions with affected people) and record review (to glean economic data for cost analysis of existing interventions)	Community members and leaders and from formal and informal health and social care providers
Micro-level: Individual-level embodied experience of the NTD-affected person	1. Life history2. In-depth interviews3. Semi-structured economic costing questionnaire	Data will be gathered from adults and children affected by mycetoma, podoconiosis and scabies, and their household-level caregivers

## Approaches to address the capacity-building aims

The 5S Foundation is designed not just to conduct research, but also to develop social science capacity across a range of local health priorities, including NTDs, through training seven PhD students and mentoring four postdoctoral research fellows. Two PhD students and one postdoctoral research fellow will be recruited in each partner country, and one PhD student and one postdoctoral research fellow in the UK. PhD students will be selected with backgrounds in social science and public health. The aim is to get social scientists to work alongside epidemiologists and clinicians who are working in the field of NTDs. Each partner country PhD student will be supervised by two UK-based supervisors and one local supervisor. All the supervisors selected have a background in social science in general and medical anthropology in particular. Few co-supervisors have a background in public health. The UK-based capacity development lead, who will be coordinating the capacity development component of the 5S Foundation, is an experienced medical anthropologist. The PhD students will conduct research into the community-level and individual-level situations elaborated earlier using a range of qualitative and ethnographic methods. The specific topic will be finalised in consultation with the supervisors and the student. However, they will explore the NTDs from different thematic perspectives. This will include the role of gender in NTD experience as women and men respond differently to illness and they are treated differently by society and family members,^46^ and ‘structural violence’, which is one way of describing social arrangements that put individuals and populations in harm's way.^47^ Structural violence is frequently perpetuated through policy, legislation and resource allocation. The PhD projects will also include ‘intersectionality’, the theory that stipulates that people are often disadvantaged by multiple sources of oppression formed by identity-makers such as gender, sex, race, class and religion.^48^

Researchers with a PhD in social science will be selected as postdoctoral fellows in partner countries and in the UK. The partner country-based postdoctoral fellows will conduct an evaluation of existing interventions. The UK-based PhD and postdoctoral research fellow will focus on the international-level situation. The PhD student will conduct a study of the ethics, justice and historical dimensions of NTDs, while the postdoctoral research fellow will explore the international policy environment around NTDs. The UK-based PhD student will be supervised by UK-based supervisors, and each postdoctoral research fellow will have a UK-based mentor to provide support to carry out the planned study and meet any capacity-development needs. The supervisors and mentors will all have a background in social science. The ultimate aim is to embed social science postdoctoral fellows and PhD students in the world of NTD research.

A skills audit will be conducted with each PhD candidate to assess their knowledge and skills gap. Following this, a training plan will be developed, tailored to each student. This training will be given through face-to-face sessions, online courses and remote training using virtual platforms. Currently, face-to-face interactions are interrupted due to the Covid-19 situation. When international travel restrictions permit, the partner country-based students will have the opportunity to visit the University of Sussex periodically and utilise on-campus facilities. Regular mentoring sessions will be arranged between the mentors and postdoctoral research fellows. Workshops and events will also be organised in the partner countries to facilitate South-South knowledge-sharing. The Research Capacity Strengthening framework for low- and middle-income countries conceptualised as being targeted at individual, institutional and societal levels will guide us in this regard.^49^

## Public engagement

In order to ensure the impact of the 5S Foundation, public engagement has been integrated into the project plan. By ‘public’ we mean stakeholders at both a national and community level. The 5S Foundation's work will be participatory and will include patients, community representatives, implementers and policy-makers. In collaboration with the Institute of Development Studies, we have developed an impact, communications and engagement strategy to ensure that we maximise the impact of the Foundation both locally and internationally. We have also formed a Strategic Advisory Board comprising national-level policy-makers in the three target countries. The Strategic Advisory Board will attend annual project meetings and engage with the discussion and planning of the ongoing project. They will review the risk register regularly and provide insight regarding the feasibility of project activities. The 5S Foundation will also support community-level public engagement activities. These will be selected based on the meso-level situation analysis. The methods of public engagement will be co-designed with community members. A dedicated Public Engagement Officer will be recruited to oversee all the public engagement activities. We will also hold an end-of-project workshop with all the relevant stakeholders.

## Process documentation

A process documentation will be carried out throughout the life of the Foundation. The aim of the process documentation will be to capture the experience and challenges of implementing the project and to give feedback to the project management team for them to consider a revision of plans if deemed necessary. The UK-based project manager will carry out the process documentation. Data sources will include the monthly project management board meetings, project core members’ meetings and a predesigned template that the partner country principal investigators (PIs) will complete regularly. Data will also be collected from the annual meeting and targeted workshops throughout the project. The process evaluation will ultimately be used to address the strategic aims of the 5S Foundation.

## Discussion

Through the research and public engagement activities detailed above, the 5S Foundation aims to change the discourse at different levels around three severely stigmatised skin conditions in Ethiopia, Sudan and Rwanda. The change in discourse will include changes to how the diseases are conceptualised, addressed and represented. These changes will be tracked through measurable outcomes. For example, at the macro-level it is expected that clear steps towards policy change will be taken in the partner countries, which may include public consultation or formation of a steering committee designed to shape policy around the relevant skin conditions in each country. At the meso-level it is expected that we will see strategies developed on social science interventions for implementers and on human resource infrastructure, in the form of more and better-trained social scientists in countries previously lacking them, which will ultimately change the practices of dealing with these conditions. It is expected that awareness will increase and stigmatisation decrease in relation to these conditions within endemic communities. At the micro-level, the expectation is to demonstrate reduced stigma and improved health and well-being experienced by affected people, reflected in several ways, including earlier presentation to health facilities.

Through rigorous research at these three levels, with the guidance of the proposed conceptual framework, the 5S Foundation plans to produce outputs applicable to policy and management-orientated decision-making for macro-level stakeholders and for meso-level audiences. The Foundation will produce guidelines for acceptable and feasible community engagement to raise awareness about NTDs. The 5S Foundation will also produce at least two PhD graduates and one experienced postdoctoral research fellow with the social science expertises to tackle NTDs in each target country. At the micro-level, the Foundation will produce an adapted tool to measure experienced stigma and life quality of the affected people. The Foundation will deliver its impact strategy through a combination of communication, dissemination and public engagement activities.

## Conclusion

We believe this project will model how to incorporate social science into addressing the problems around NTDs in particular, and many other important global health problems of today in general.

## Data Availability

As this is a protocol paper, no primary data has been used.
